# An Adaptive Energy Saving Algorithm for an RSSI-Based Localization System in Mobile Radio Sensors

**DOI:** 10.3390/s21123987

**Published:** 2021-06-09

**Authors:** Adam Olesiński, Zbigniew Piotrowski

**Affiliations:** Communications Systems, Faculty of Electronics, Military University of Technology, 00-908 Warsaw, Poland; zbigniew.piotrowski@wat.edu.pl

**Keywords:** automatic transmitter power control (ATPC), short range devices (SRD), received signal strength indicator (RSSI), wireless radio sensors, indoor localization

## Abstract

In localization systems based on the emission of reference radio signals, an important issue related to the reliability of sensor operation is the problem of operating time and power of the emitted reference radio signal. There are many localization methods that have proven useful in practice and that use a reference radio signal for this purpose. In the issue of determining the location of radio emitters, various radio signal propagation models are used to determine the effective range and distance of the sensor-receiver from the radio emitter. This paper presents an adaptive power control algorithm for a transmitter, as a reference emitter, operating in power-saving mode. An important advantage of the presented solution is the adjustment of the localization system accuracy at the assumed level of energy radiated by radio emitters based on the RSSI signal received power estimation.

## 1. Introduction

There are many publications on localization using the RSSI (received signal strength indicator) method and a fusion of localization methods, including RSSI, enabling the localization of moving radio effectors, e.g., indoors. Ref. [1] describes the investigation of the mere presence and movement of people as one of the main factors causing changes in the RSSI reading and position estimation using various methods. Refs. [2,3,4] describe the influence of the antenna (including switched beam antennas) on the positioning system using the RSSI method. The very problem of the error caused by the RSSI, which affects the accuracy of position estimation, is often the subject of publications on in-depth analyses [5], attempts to correct and predict this error [6], and the synthesis of new optimal location algorithms [7,8,9,10] including the use of neural networks [11,12]. The algorithm, proposed in [10], seems to be of particular interest, where, according to the authors, the results of the location estimate have improved by 80.97% in the office room and 67.51% in the corridor, respectively, compared to the traditional RSSI positioning algorithm. In the case of the possibility of using multi-sensor location systems inside buildings, the use of convolutional neural networks (CNN) brings good results. According to the authors of [12], the system consisting of 13 radio sensors connected to a neural network with the proposed structure shows the high accuracy of position estimation (97.92% with an error of 2.8%) of a human moving in a complex office environment, without the need to perform complicated calibration. A good idea of improving the accuracy is also the fusion of several methods, such as in [13,14,15], mainly AOA (angle of arrival) and RSSI.

There are also publications on optimal automatic transmitter power control (ATPC) algorithms such as [16], including those showing the influence of the algorithm on the position estimation accuracy [17] and showing the need to use more transmitter power at longer distances to reduce the estimation error [18]. Unfortunately, the number of studies on the use of ATPC in position estimation and confirmed by practical experiments is still quite small. In [19], the authors propose the ATPC algorithm with feedback making the transmitter power dependent on the LQI (link quality indicator). The authors pay attention to the existence of serious gaps between the existing theory work and in situ practice. During the scientific research preceding the study described in the paper, there was no explicitly described algorithm for the automatic control of the transmitter power operating on the qualitative indicator—error of distance estimation—enabling a compromise between energy consumption and position estimation error depending on the assumed work scenario of the mobile radio effector. Moreover, most publications mentioned the obvious influence of radio channel interference on the RSSI index, but no explicit relationship was found or attempts to deliberately inject spurious signals with known parameters during the operation of the ATPC algorithm. Therefore, in this paper, it was decided to explain the influence of transmitter power on the accuracy of distance estimation in the RSSI method and then propose a simple ATPC algorithm containing sensor feedback.

## 2. Location of Radio Sensors

**Figure 1:**
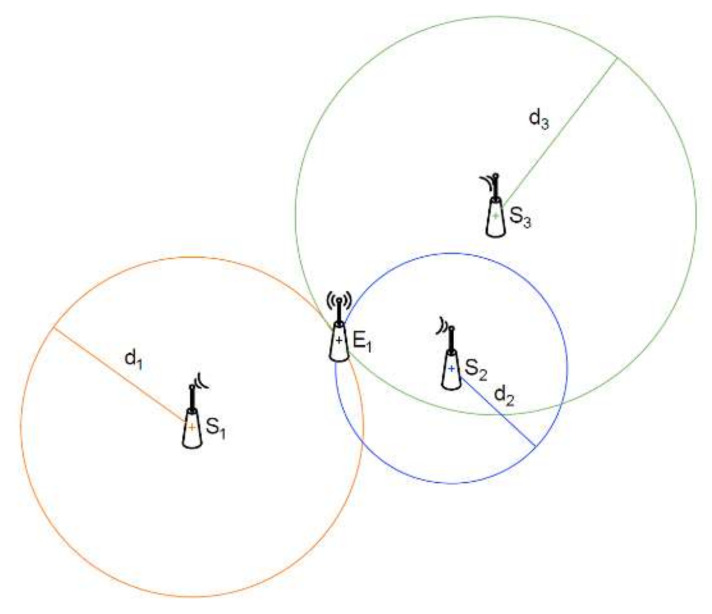
Illustration of the effector location by radio sensors, using trilateration.

Wireless radio sensors are generally small devices for a variety of purposes. They are most often used for surveillance or remote data acquisition. They are also part of larger wireless sensor networks (WSNs) or the Internet of Things (IoT). They are used in industry, construction, security, smart buildings, or smart city infrastructure. For some time now, they have also been used for indoor localization, for example. Radio effector localization by radio sensors can be done by TOA (time of arrival), TDOA (time difference of arrival), AOA, COO (cell of origin), or by RSSI. The TOA method requires accurate time synchronization of all radio devices involved in the localization. In the TDOA method, accurate time synchronization is not required; however, this method requires expensive solutions to measure small time differences (in the order of picoseconds) occurring at small distances. In the AOA method, the RF sensor must be equipped with a solution to measure the angle of arrival of the electromagnetic wave (e.g., an antenna array). The COO method only states which cell the radio effector is currently in the range. For indoor use, the RSSI-based localization method is the most optimal. It is less accurate, but since the RSSI indicator is readable in essentially any integrated radio transceiver (SRD, WIFI, Bluetooth, ZigBee, and others), it is commonly used for this purpose. This method estimates the distance based on the received signal power and the selected propagation model. Then, using trilateration [20,21,22,23,24,25,26] or multilateration and the known coordinates of the cooperating stations, the location of the localized radio effector can be determined, as shown in Figure 1.

However, the key is to accurately determine the radius d. Typically, for this purpose, the Log-distance path loss model is used [20,21,22,23,24,25,26,27,28,29,30,31,32,33,34,35] in which the power over distance is *d*, described by the formula:

(1) $$P\left(d\right)\left[\mathrm{dBm}\right]{=P}_{d0}\left[\mathrm{dBm}\right]-10\cdot n\cdot \log_{10}\left(\frac{d}{d_{0}}\right)$$

where *n* is the attenuation exponent of the electromagnetic wave and typically takes values in the range of <2;4>, *d* is the distance between the receiver and transmitter antennas. *P_d_*_0_ is the power measured at a distance *d*_0_ from the transmitter and depends on the transmitter power P_TX_, transmitter antenna gain G_TX_, and electromagnetic wave attenuation L over distance *d*_0_:

(2) $$P_{d0}\left[\mathrm{dBm}\right]{=P}_{\mathrm{tx}}\left[\mathrm{dBm}\right]{+G}_{\mathrm{tx}}\left[\mathrm{dB}\right]-L\left(d_{0}\right)\left[\mathrm{dB}\right]$$

Finally, the power at the receiver input gates depends on *P(d)* and the receiver antenna gain G_TX_.

(3) $$P_{P}\left[\mathrm{dBm}\right]{=G}_{\mathrm{rx}}\left[\mathrm{dB}\right]+P\left(d\right)\left[\mathrm{dBm}\right]$$

Therefore, the ratio of the radius *d* to *d_0_* is:

(4) $$\frac{d}{d_{0}}=10^{\frac{\mathrm{RSSI}-G_{\mathrm{rx}}-P_{\mathrm{tx}}-G_{\mathrm{tx}}+L\left(d_{0}\right)}{-10\cdot n}}$$

Which, with the assumed *d*_0_ = 1m and measured *P_d_*_0_, can be reduced to the radius value *d* [21]:

(5) $$d\left[m\right]=10^{\frac{\mathrm{RSSI}-G_{\mathrm{rx}}-P_{d_{0}}}{-10\cdot n}}$$

**Figure 2:**
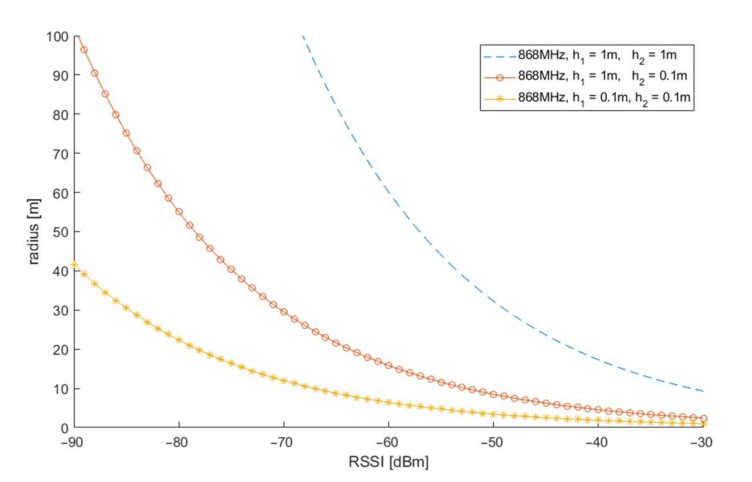
Dependence of the distance between radio sensors on RSSI (received signal strength indicator) for different antenna heights (h_1_ and h_2_).

Figure 2 shows the dependence of the radius d on the RSSI value for different antenna heights (relative to the antenna’s ground, in meters) resulting from Equation (5). Empirically, a value of n = 3.7 was chosen as a good representation of wave attenuation for low-lying indoor shortened antennas. The lower the antennas of radio effectors and sensors are, the smaller the possible range of radius d estimation for fixed receiver sensitivity.

## 3. Influence of Transmitter Power on the Location Accuracy of the RSSI Method

The consideration of the effect of transmitter power on the RSSI radius estimation error makes sense in portable radio effectors, where the priority is to conserve power to maximize battery life through automatic transmitter power control (ATPC) [21]. The effect of transmitter power on location accuracy in the RSSI method is not apparent directly and needs to be clarified, specifically, what exactly the received power indicator is. It is a measure of the power present in the radio channel. It means that the power indicated by RSSI consists of the received useful signal, noise, interferences, and disturbances in the channel, as well as products of intermodulation occurring on non-linear elements of the RF transceiver path (especially important in zero-IF receivers with low dynamic parameters). Thus, it can be written that:

(6) $${\mathrm{RSSI}=P}_{P}{+P}_{N}{+P}_{I}$$

Naturally, remembering that, when adding the power in dBm, one should use an expression like the following:

(7) $$P\left[\mathrm{dBm}\right]={10\log}_{10}\left(10^{\frac{P_{1}\left[\mathrm{dBm}\right]}{10}}+10^{\frac{P_{2}\left[\mathrm{dBm}\right]}{10}}\right)$$

where *P_P_* is the power of the useful signal, *P_N_* is the power of the noise in the channel, and *P_I_* is the power of the interfering signals. According to the radio link energy balance Formulae (1,2), as the transmitter power decreases, the power at the receiver terminals decreases and, therefore, the SINR (signal to interference plus noise ratio) decreases:

(8) $$\mathrm{SIN}R=\frac{P_{P}}{P_{I}{+P}_{N}}$$

**Figure 3:**
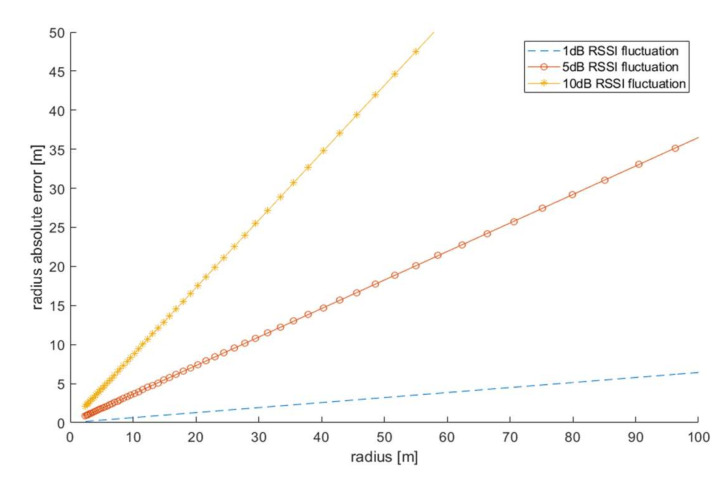
Absolute error of the distance measurement for different values of the RSSI fluctuation.

For steady-state conditions, when *P_I+N_* = const, the effect of transmitter power is negligible as long as SINR is still large enough (*P_I+N_* << *P_P_*). However, in real radio conditions, when many devices work in a channel (e.g., BLE, Wi-Fi, SRD, but also not only radio, e.g., microwave ovens), collisions and interferences occur, temporary and periodical interferences appear, and it results in an increase in random errors in the RSSI reading (fluctuations), despite still satisfactory PER (Packet Error Rate), allowing to maintain the link. The error introduced by interference is difficult to model, as it is a direct result of the radio environment in which radio sensor localization is performed. We can write the absolute error of distance measurement itself in the RSSI method as:

(9) $$\Delta d=\left|d-10^{\frac{\mathrm{RSSI}-G_{\mathrm{rx}}-P_{d_{0}}}{-10\cdot n}}\right|$$

where *d* is the actual distance between the receiver and transmitter antennas. The effect of the RSSI error on the absolute error of the estimated distance is shown in Figure 3. As can be seen, the effect of RSSI fluctuations [23,24,25,26,27] on the absolute ray error value ∆d is directly dependent on the distance between the receiver and transmitter and, obviously, on the error contributed by RSSI.

**Figure 4:**
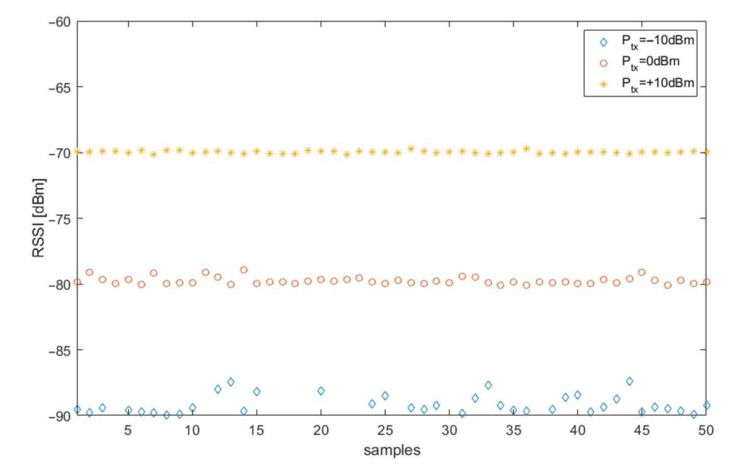
Simulation of the received RSSI values for different transmitter power values when operating on a radio channel.

Figure 4 shows a simulation of the effect of transmitter power on the fluctuations [31] of RSSI when taking into account noise and interference from other stations operating in the same channel. Transmitter power was decremented in 10 dB increments. There was a noticeable increase in RSSI fluctuation as the transmitter power decreases.

## 4. Automatic Power Control Algorithm

**Figure 5:**
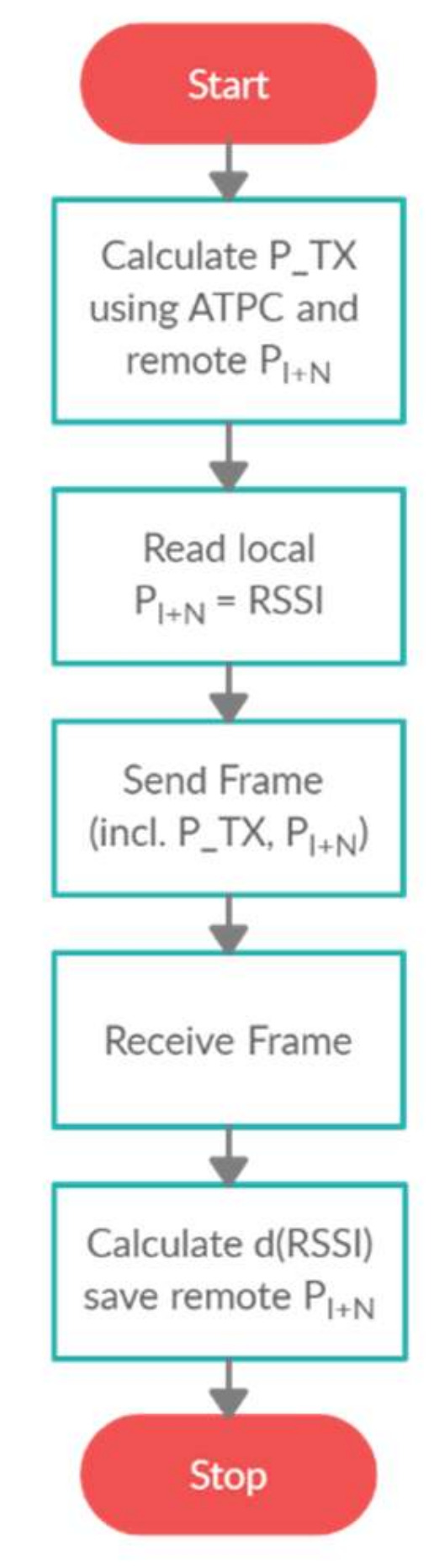
Simplified algorithm for data exchange between radio effector and sensor.

In order to synthesize the ATPC algorithm [19], the necessary variables must be defined to properly determine the transmission power. The maximum possible error level ∆RSSI for the set error value ∆*d* and the current sensor distance d have to be specified. In order to calculate the minimum transmitter power P_TX_ based on ∆RSSI, a known value of *P_I+N_* on the receiving side is needed. One can attempt to estimate the remote *P_I+N_* value based on local radio conditions and the distance between the effector and the sensor; however, a simpler way is to assume a two-way communication between the effector and the sensor to exchange *P_I+N_* data and mutual initial distance estimation using high transmission power to initialize the variables. A simplified algorithm for data exchange between radio devices [20] is shown in Figure 5. Therefore, the described algorithm was best suited for solutions that localize the effector movement continuously, e.g., by localizing human movement inside a closed object in real time, based on the fusion of RSSI and COO methods.

**Figure 6:**
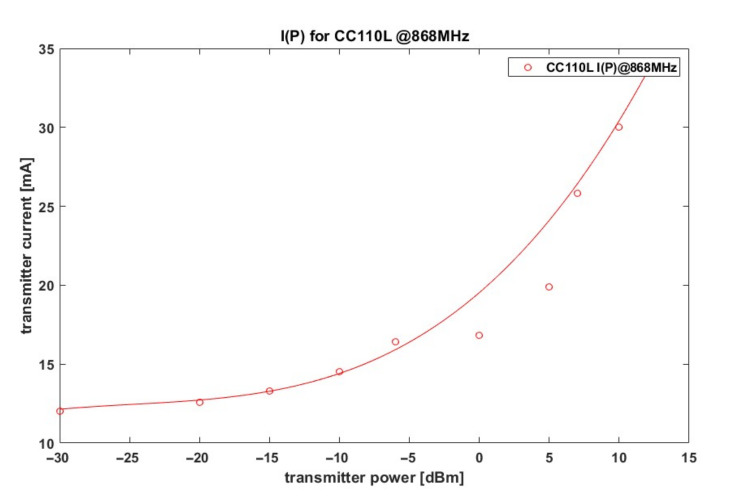
Dependence of the current consumed by the CC110L for different transmitter powers.

The ATPC algorithm in portable, battery-powered, or battery-operated radios is designed to maximize the operating time of the device while having as little impact as possible on location accuracy. The operating time of the RF effector could be extended by reducing the transmitter power and thus reducing the current drawn by the RF transceiver. Figure 6 shows the dependence of the current drawn on the transmitter power for the popular RF transceiver CC110L, manufactured by Texas Instruments, TX, USA [33].

**Figure 7:**
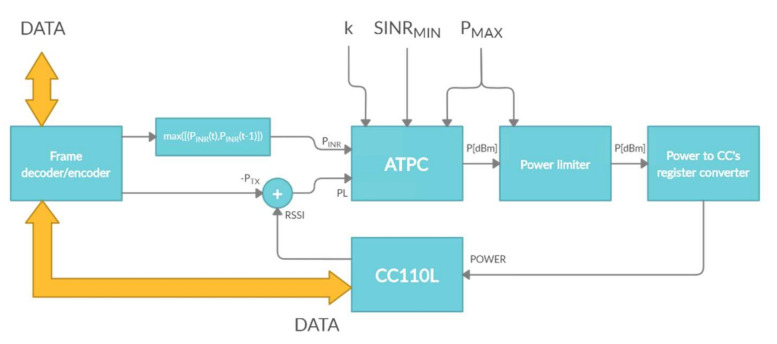
Block diagram of the automatic transmitter power control (ATPC) algorithm working with CC110L.

Reducing the transmitter power, however, consequently reduced the accuracy of the distance measurement taken. This means that the ATPC algorithm used in the sensor localization system based on the RSSI method must provide a kind of compromise between energy savings and localization accuracy. Therefore, a mathematical description of the core of the tracking algorithm with the ability to adjust the resulting power (accuracy) with the ***k*** factor was proposed:

(10) $$P\left[\mathrm{dBm}\right]=k\cdot P_{\mathrm{MAX}}+\left(1-k\right)\cdot \left(P_{\mathrm{INr}}{+\mathrm{SIN}R}_{\mathrm{MIN}}+\mathrm{PL}\right)$$

where *k* ∈ <0;1>, *P*_MAX_ is the highest transmitter power that can be set while providing the highest distance estimation accuracy. *P*_INr_ is the remotely reported channel interference power at the receiver side. SINR_MIN_ is a constant for the entire system of identical RF sensors, defining the minimum ratio of signal power to noise power and interfering signals, ensuring a sufficiently low PER. PL is the attenuation of the electromagnetic wave described as:

(11) $$\mathrm{PL}\left[\mathrm{dB}\right]{=P}_{\mathrm{TXr}}-{\mathrm{RSSI}}_{l}$$

where RSSI*_l_* is the channel power value when receiving a radio frame and P_TXr_ is the remotely reported transmitter power used in the transmission. On analyzing Equation (10), it could be seen that for *k* = 0, the resulting transmitter power would maintain the transmission and distance measurement for the remotely measured *P*_INr_ for the discretized instant t-1, at which a frame was received from the remote sensor. On the other hand, at time t, when the radio effector transmits a frame to the sensors, the conditions in the channel at the receiving side would be different, and the probability of correct reception and distance estimation with satisfactory error decreased. Figure 7 shows the block diagram of how the ATPC algorithm works with the CC110L chip [33].

Of course, the output power must be limited to the maximum output power of the transmitter:

(12) $$P_{\mathrm{TX}}=\left\{\begin{array}{c} P_{\mathrm{est}}{\mathrm{when}\;P}_{\mathrm{est}}{<P}_{\mathrm{MAX}}\\ P_{\mathrm{MAX}}{\mathrm{when}\;P}_{\mathrm{est}}⩾P_{\mathrm{MAX}} \end{array}\right\}$$

## 5. Test Scenario

**Figure 8:**
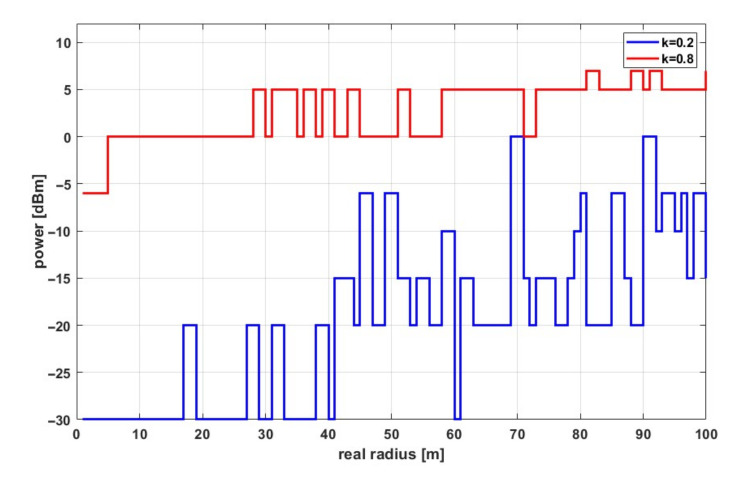
Transmitter power depending on the actual distance for different *k*-factor.

The study of the proposed ATPC algorithm was performed by simulating the communication of an effector with a radio sensor, moving away from each other by a fixed distance ∆R in time steps ∆t. 2000 simulations were performed for different values of the coefficient *k*. Real propagation phenomena were considered during the study using a two-ray ground-reflection model. Interference on the receiving side was simulated with a random variable with a normal distribution in the dBm domain, with a mean value of −110 dBm [25]. The parameters of the radio path are shown in Equation (13).

(13) $$\left\{\begin{array}{c} f_{0}=868.0\;\mathrm{MHz}\\ \Delta f=18\;\mathrm{kHz}\\ \mathrm{DR}=38.4\;\mathrm{kBaud}\\ \mathrm{MOD}=\mathrm{GFSK}\\ G_{\mathrm{TX}}{=G}_{\mathrm{RX}}=0\;\mathrm{dBi}\\ h_{\mathrm{TX}}{=h}_{\mathrm{RX}}=1\;m\\ P_{\mathrm{MAX}}=+10\;\mathrm{dBm}\\ D=10\;\mathrm{ms}\\ T=1\;s \end{array}\right\}$$

where *f*_0_ is the fundamental frequency, ∆f is the frequency deviation, DR is the data rate, MOD is the modulation used, *h*_TX_ and h_RX_ are the height above ground of the remote and local sensor antennas, D is the duration of the radio frame (depends on the amount of data and DR), T is the transmission repetition period, and *G*_TX_ and G_RX_ are the antenna gains on the remote and local sensor side, respectively. The result of the ATPC algorithm in the presented test scenario is shown in Figure 8.

**Figure 9:**
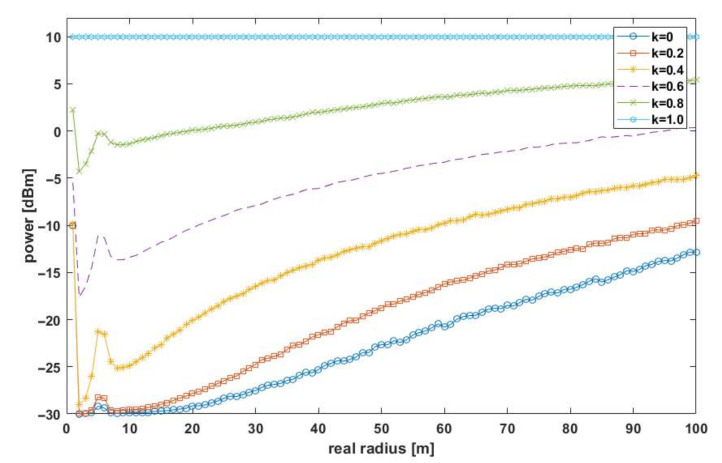
The average power of the transmitter for different values of the *k*-factor.

The plot showed the resulting transmitter power determined by the running ATPC algorithm as a function of the actual distance between the sensors for two different values of the coefficient *k*. The stepped nature of the graph was due to a predefined set of transmitter power values that could be set in the CC110L chip register [33] (−30 dBm, −20 dBm, −15 dBm, −10 dBm, −6 dBm, 0 dBm, +5 dBm, +7 dBm, +10 dBm). This means that the resulting power value from the algorithm was rounded up to a settable value. In turn, Figure 9 shows the transmitter power values averaged from all simulations for different values of the coefficient *k*.

**Figure 10:**
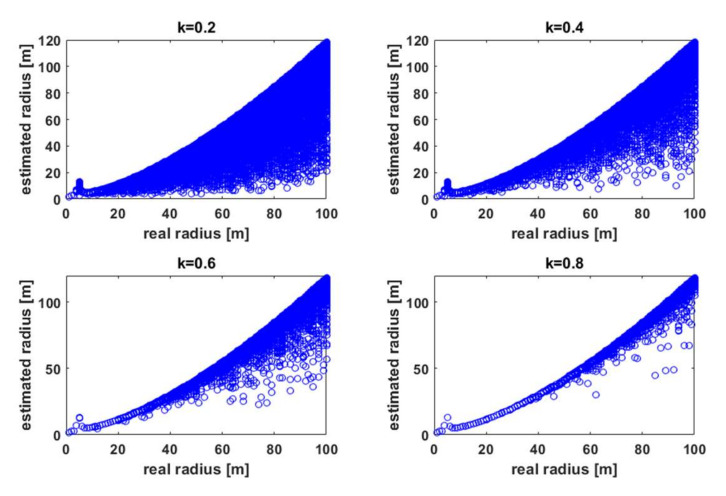
Values of the distance estimated as a function of the real distance for different values of the *k*-factor.

It can be seen that for both short and long distances between the effector and the sensor, the difference in transmitter power for different *k* was roughly proportional. This means in simple terms that k_TX_ ~ SINR_RX_. Because of this relationship, the effect of the *k* coefficient on localization accuracy using the RSSI method was analyzed [26]. Figure 10 shows the estimation results (as a set of points) of the effector-sensor distance for the running ATPC algorithm (Equation (10)) as a function of distance for different values of the *k* coefficient for AWGN channel interference described by a normal distribution with a constant mean of −110 dBm [25].

**Figure 11:**
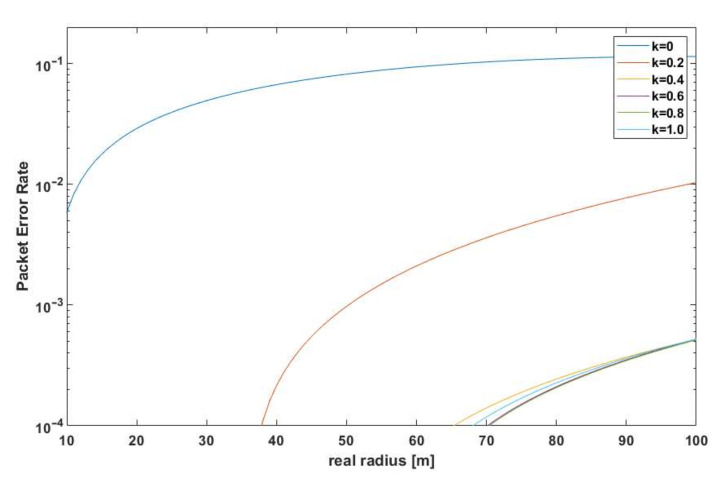
PER values for different *k*-factor values.

A clear effect of fluctuating RSSI values on the estimated distance was observed [26]. The number of estimates with a large absolute error was greater the lower the power of the useful signal at the receiver terminals (lower *k*). The error also increased with distance (according to Equation (9)). The estimates did not take into account the case when SINR was too low to decode the frame correctly. For simulation purposes, such cases were detected, and the dependence of PER (Packet Error Rate) on *k* [28,29] was plotted (Figure 11).

**Figure 12:**
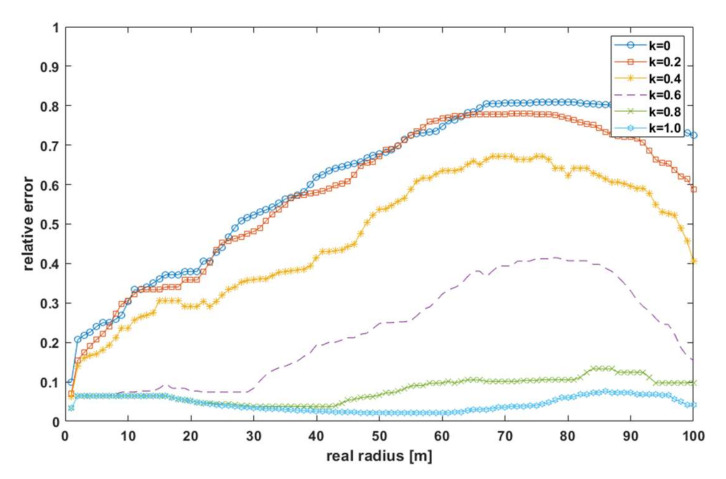
The maximum relative error of the distance estimation for different values of the *k* coefficient.

For this test scenario, the proposed algorithm for *k* = 0 was basically not applicable—the PER was too high, while for *k* = 0.2, abstracting from the estimation error, it could be successfully applied in a limited distance range. In order to analyze the effect of the ATPC algorithm performance on the localization accuracy, the relative error *δ*_d_ [31] of the distance measurement as a function of the actual distance is shown in Figure 12.

This error was determined from the estimates shown in Figure 10 by finding, for each distance and factor *k*, the largest absolute error ∆d_MAX_ in the simulation group and then converting the relative error according to the formula:

(14) $$\delta_{d}=\frac{{\Delta d}_{\mathrm{MAX}}}{d}$$

where *d* is the actual distance between the effector and the sensor. By analyzing Figure 12, it could be seen that the relative error increased with distance and with decreasing the coefficient *k*. The maxima appearing between the actual distances of 70–90 m were due to the simulation methods adopted—the actual power at the receiver terminals was calculated using the two-ray ground-reflection model, while the RSSI estimated distance was calculated using the Log-distance path loss. The models sized up relative to each other; hence, in Figure 12 above, certain distances of the relative error decreased.

For further analysis, the effect of the ATPC algorithm on effector energy conservation was considered. The energy saving was proportional to the saving of the load consumed during data transmission. The amount of charge for a single frame with duration *D* and transmitter current *I*(*P*_TX_) is:

(15) $$C\left[\mathrm{mAh}\right]=\frac{I\left(P_{\mathrm{TX}}\right)\cdot D}{3600}$$

**Figure 13:**
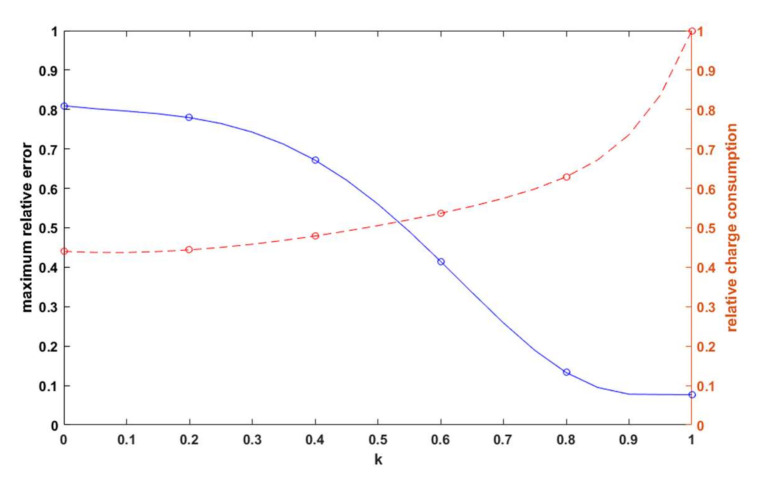
The maximum relative error of the distance estimation and the relative charge consumption as a function of *k*.

Through the simulations, data was collected on the power used for data transmission for each case. After converting the transmitter power to the current, the total charge consumed for each *k* was summed, which was summarized as the ratio of the total charge for a given *k* to charge for *k* = 1, along with the relative error of the distance measurement in Figure 13.

**Figure 14:**
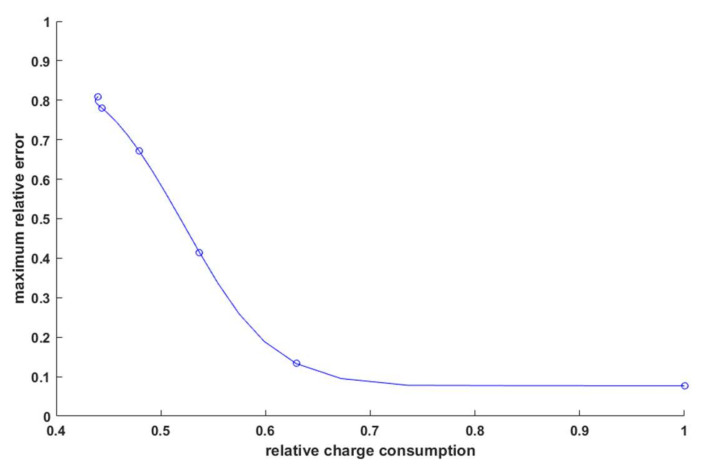
Dependence of the maximum relative error of distance estimation on the relative load consumption.

On analyzing Figure 13, three characteristic areas could be identified. For *k* ∈ (0.8;1 >, a large change in charge draw of 37% resulted in a decrease in the relative error of only 5.6%. The work of the algorithm in this area was rather unreasonable in terms of energy savings. For *k* ∈ <0;0.4>, a 3.92% increase in total charge taken caused a 13.77% decrease in the relative error. However, it should be noted that even for *k* = 0.4, the maximum relative error of the distance estimate was still about 67%. This was, of course, the worst possible case resulting from taking the maximum error values from the waveforms plotted in Figure 11 (for real distances from about 60 to 80 m). Area *k* ∈ (0.4;0.8> was a quasi-linear relationship in which, as the charge collection decreased by 15%, the relative error of the distance estimate increased by 53.9%. This gave an increase in the maximum error of about 0.036 for each 1% of charge saved. Such proportionality gave the possibility to modulate the error in such a way as to achieve the maximum operating time of the sensor while maintaining a satisfactory distance estimation error. In order to better illustrate the relationship, the relative estimation error and charge savings were related through the parameter *k* and are shown in Figure 14.

**Figure 15:**
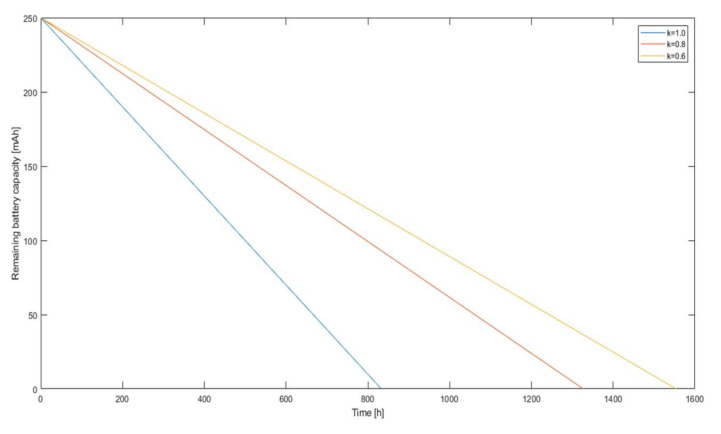
Battery charge remaining as a function of time for different *k*-factor values.

As can be seen on the cut-off axis between 0.736 and 1, the relative error did not change and was approximately 0.077. A point of 0.736 or 26.4% load savings with no increase in the estimation error corresponded to *k* = 0.9. In contrast, *k* = 0.8 was an inflection point that seemed to be an appropriate compromise between the relative error and energy savings. In the case of a small radio sensor equipped with a battery of an example capacity of 250 mAh, according to the data in Equation (13), the dependence of battery discharge as a function of time was plotted for different values of the coefficient *k* in Figure 15. Assuming that the transmitter was the only device drawing power from the battery, for *k* = 1, the device would work for 34 days and 17 h; for *k* = 0.8*,* it would work for 55 days and 4 h; and for *k* = 0.6*,* it would work for 64 days and 18 h, with the difference in the error between *k* = 1 and *k* = 0.8 being about 5.6% and the gain in operating time being about 21 days. The difference in error between *k* = 0.8 and *k* = 0.6 was already over 21%, and the gain in runtime was only another 9 days, confirming that *k* = 0.8 was the best compromise for the chosen test scenario.

## 6. Hardware Implementation

It was decided to implement the ATPC algorithm in the microcontroller, using the bare metal C programming language, and checking its performance. For this purpose, two NUCLEO-F303RE evaluation boards, manufactured by STMicroelectronics, Switzerland [36] containing the STM32F303RET6 microcontroller [37] were prepared. Although the algorithm was simulated for the CC110L chip [33], it was decided to check its operation for another RF transceiver—RFM95W, manufactured by HopeRF, China [38]—which supports the chirp spread spectrum using LoRa modulation. This integrated module had a power regulation dynamic of 18 dB, in increments of 1 dB. In accordance with the requirements of the algorithm, two-way communication with data exchange was implemented. Communication was initiated by the RF effector with an implemented ATPC algorithm, while the RF sensor transmitted with constant power, informing the effector about the current noise and interfering signals power in the channel—PINR. The transceiver configuration parameters are shown in (16).

(16) $$\left\{\begin{array}{c} f_{0}=868.0\;\mathrm{MHz}\\ \mathrm{BW}=500\;\mathrm{kHz}\\ \mathrm{MOD}=\mathrm{LoRa}\\ \mathrm{CR}=4/5\\ \mathrm{Sf}=128\mathrm{chips}/\mathrm{symb}\\ P_{\mathrm{MAX}}=+23\;\mathrm{dBm}\\ D=100\;\mathrm{ms}\\ T=1\;s \end{array}\right\}$$

where CR is the cycling coding rate, affecting the forward error detection and correction, and SF is the Spreading Factor, which determines the amount of chips per symbol. SINR_MIN_ for the algorithm was set to + 7 dB, although the processing gain in LoRa allowed reception with an SNR lower than 0 dB [38]. The maximum power +23 dBm was the highest value of the transceiver output power—in order to carry out tests in the real environment, some measurements were performed with a 30 dB attenuator attached to the antenna connector, so the maximum power at the effector antenna terminals during some measurements was −7 dBm.

**Figure 16:**
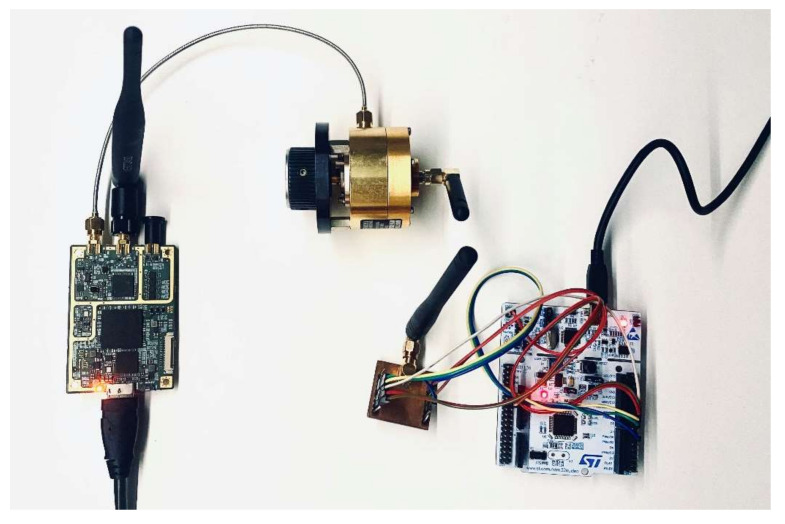
Measure stand: radio sensor and device for injecting disturbances and spectral observations —USRP-B200mini with an attached adjustable attenuator.

Figure 16 shows the measurement stand from the side of the radio sensor. From the left, it was an SDR (Software Defined Radio) transceiver—USRP-B200mini, manufactured by Ettus Research, TX, USA [39]—which was used to observe the radio spectrum and perform selective channel jamming. A variable attenuator was attached to the TX path of the SDR, ensuring a good matching and power output adjustment independent of the SDR software settings. An antenna was attached to the variable attenuator, which was located close to the RFM95W module antenna [38]. The module itself was attached to the NUCLEO-F303RE [36] evaluation board, in accordance with the technical documentation.

The measurements were performed in a closed room of approximately 40 m^2^, in which the RF sensor and the RF effector were placed on the floor at a distance of approximately 4 m between them, and the antennas at a height of approximately 10 cm, the previously recorded value of path loss PL = 88dB for 1m distance between them, with a 30 dB attenuator used in the effector’s rf path, in order to use the log-distance path loss model [20,21,24,26,32,35] to estimate the relative error. The value of the coefficient n = 2.12 was selected empirically. A total of 244 measurements were collected for different values of the *k*-factor.

**Figure 17:**
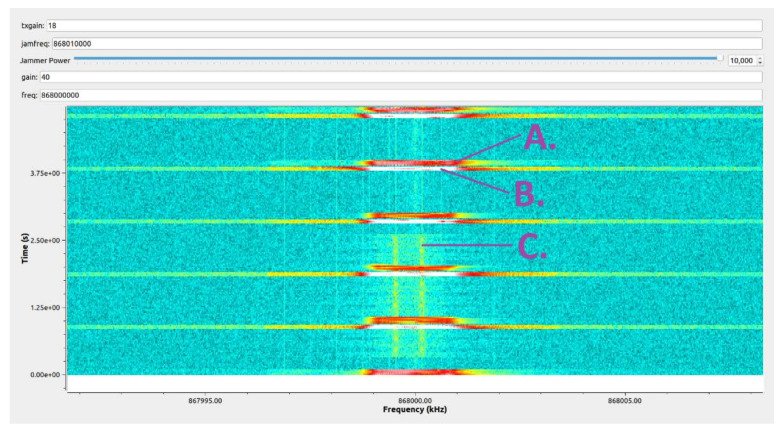
RF spectrum histogram during tests.

Figure 17 shows the waterfall histogram of the radio electromagnetic spectrum at the bench for the center frequency of 868.0 MHz. Signal A was a radio emission from the effector (T = 1 s), signal B was a response from the sensor containing a report on the RSSI value of the received A-frame and the RSSI value just before sending the B-frame (required for the correct operation of the ATPC algorithm), i.e., specifying the level of interfering signals and noise—PINR. The C signal was a cyclically switched on and off interfering-noise-modulated signal generated by the SDR transceiver using the GnuRadio software version 3.9.0.0-git. The intensity of the histogram colors depended on the signal strength at the terminals of the SDR receiver. Signal C was intended to simulate a volatile situation in the radio channel, including signals from different stations.

**Figure 18:**
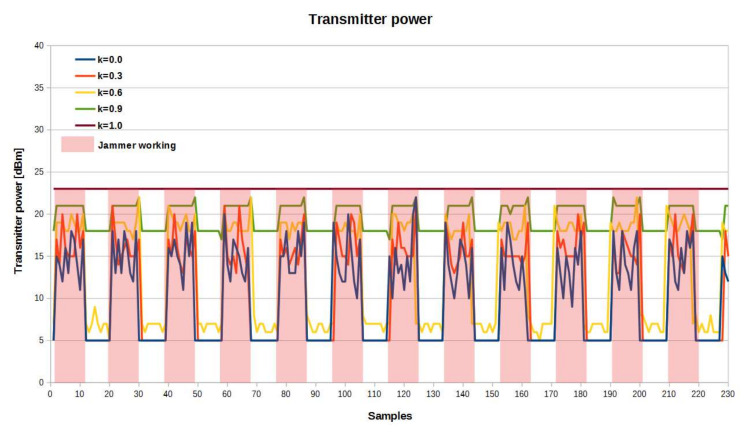
Transmitter power with and without jamming for different *k* values.

Figure 18 shows the dependence of the transmitter power value change of the RF effector for subsequently sent radio frames. The red color indicates the area in which RF jamming occurred. It could be observed that the algorithm adapted to the transmitter power to keep a minimum SINR for the receiver for *k* = 0 (the lowest possible energy consumption), also for the moments when the jammer worked. For *k* = 1 (the highest location accuracy), the algorithm selected the maximum transmitter power, regardless of the channel situation.

**Figure 19:**
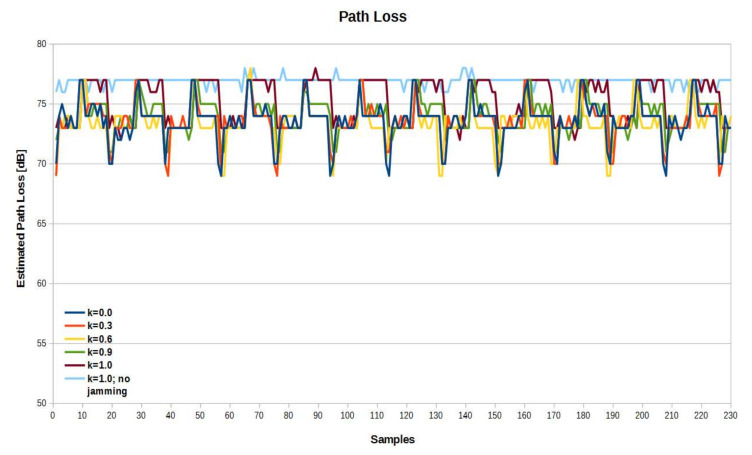
Path loss values without attenuator in the effector antenna path.

Figure 19 shows the path loss value chart calculated on the basis of the received RSSI by the sensor without the applied attenuator in the effector antenna path. The calculated path loss value depended on the reported RSSI value of the sensor side and the effector transmitter power, according to Formula (11). Due to the clarity of the chart, jamming moments were not indicated in a different color. The jammer power value was selected empirically to keep the link stable while observing clear RSSI fluctuations. According to the theoretical predictions, other radio devices (jammers) operating in the same radio channel affected the value of the RSSI returned in the locating RF sensor, namely they caused a false reduction of the path loss value and, consequently, the reduction of the estimated distance between the effector and the sensor. There was a big difference in RSSI fluctuations for *k* = 1.0 with jamming and no jamming turned on. For *k* < 1.0 with jamming on, the differences for the individual coefficient values were less significant but still present, the difference between *k* = 0.9 and *k* = 0.6 was particularly noticeable.

**Figure 20:**
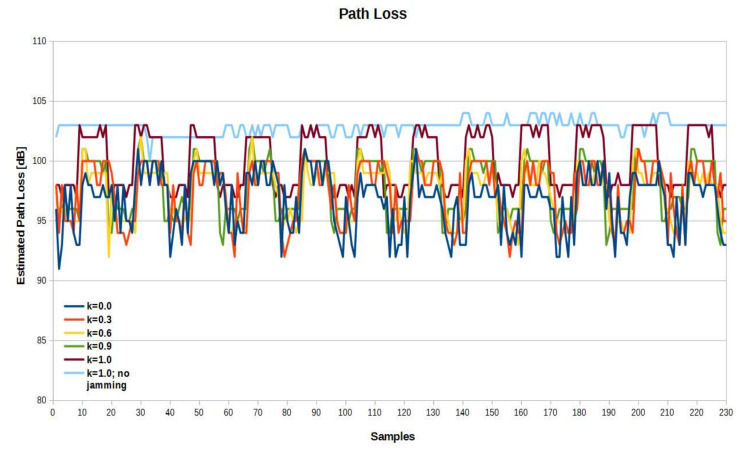
Path loss values with 30dB RF attenuator in the effector antenna path.

Figure 20 shows the path loss value calculated on the basis of the received RSSI by the sensor, with a 30 dB attenuator in the effector antenna path. In this case, a significant decrease in the level of the useful signal and re-selection of the appropriate jamming signal level resulted in an increase in differences in the maximum absolute error for different values of *k*.

In Table 1, the preprocessed data of the 30dB attenuator experiment is shown. Headings min(D) and max(D) stand for, respectively, minimum and maximum estimated distance between radio effector and sensor. MSE stands for the mean square error, RMSE means root mean square error.

**Figure 21:**
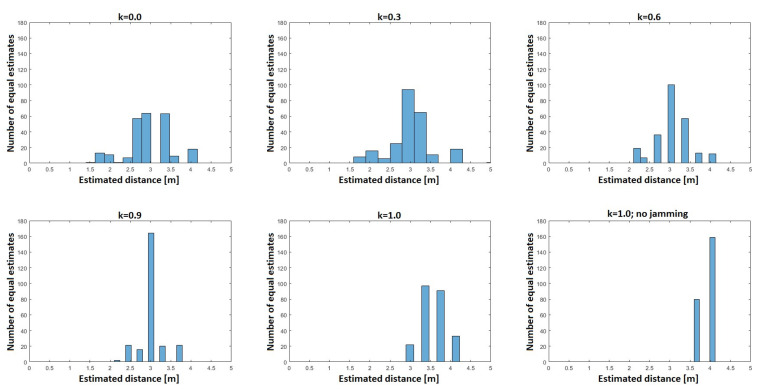
Histograms of distance estimation for various *k* values.

**Figure 22:**
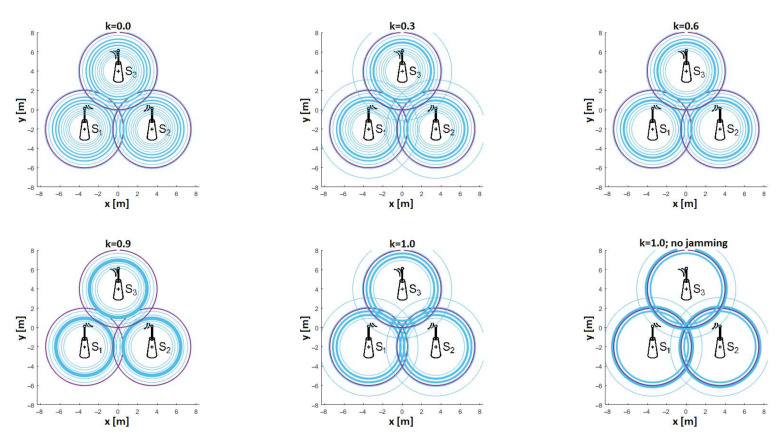
Visualization of the trilateration for the same distance estimation values for all radio sensors.

Based on the measurement results with a 30 dB attenuator, it was decided to estimate the distance values for each path loss value. Then, on the basis of the obtained results, histograms were prepared for different values of *k* and shown in Figure 21. As can be seen already for *k* = 1.0, with jamming, the maxima of the histograms were below the real value, 4 m; however, as the coefficient k decreased, the number of equal estimates began to decrease, which was illustrated by the increased randomness, and thus, an increase in the localization error. Additionally, the imaging of trilateration made of three radio sensors was performed for the case when each of them estimated exactly the same distance value on the basis of the path loss. The imaging is shown in Figure 22. The violet line was the actual circle in which the radio effector is located. The thickness of the blue line represented the number of identical estimates.

**Figure 23:**
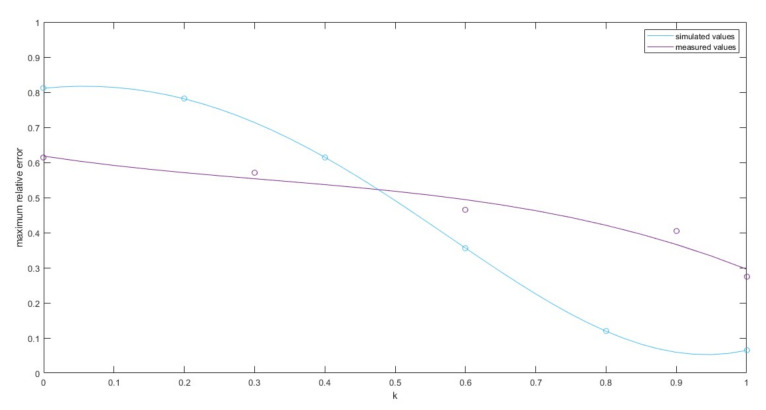
Comparison of simulated and measured maximum values of the relative error in the distance estimation for different *k*-factor values.

As can be seen, as the value of the *k* coefficient decreased, the number of distance estimates closer to the sensors increased, and the number of estimates oscillating around one distance value decreased. This means that the *k*-factor of the ATPC algorithm had an impact on the localization accuracy in the RSSI method.Additionally, the maximum values of the absolute errors of the distance estimates for various values of *k*, determined from measurements, are shown in Figure 23, with the theoretical values derived from the simulation in Figure 13.

The downward trend of the error with the increase in the *k* coefficient was maintained. However, the difference between the maximum and minimum error was much smaller for the actual measurements than for the simulation. This effect might be caused by many factors, ranging from simulation assumptions, through verification of the algorithm’s operation in a real radio environment using a different transceiver chip, with different modulation and bandwidth than used as the input data for the simulation, or the measurement for only one distance at unfavorable radio conditions (low-placed antennas, small distance between the sensor and the effector, small room in which the measurement was performed). Nevertheless, the influence of the *k* coefficient during the operation of the ATPC algorithm in the RF effector on the accuracy of distance estimation in real radio conditions was confirmed.

## 7. Summary and Opportunity for Development

This paper presented an algorithm for automatically adjusting the transmitter power of a radio sensor working to save energy. The effect of transmitter power on localization accuracy in the RSSI method was described. A test scenario was proposed to verify the performance and to outline the compromise between energy savings and localization accuracy. The given test scenario was one of many possibilities of using a portable sensor, and it should be kept in mind that the propagation environment in which the ATPC algorithm was used had a great influence on the accuracy of the distance estimation between the sensors.

The adjustability between accuracy and savings was implemented with a single variable *k*, where *k* = 0 represents the maximum energy savings and *k* = 1 represents the maximum localization accuracy. For the assumed propagation environment included in the scenario, a compromise solution was found to be the coefficient *k* = 0.8. Under other conditions, one would need to precede the running of the ATPC algorithm with an analysis of the propagation environment. This was a resource- and time-consuming task. As a development opportunity, one could try to synthesize an algorithm for automatic coefficient selection based on the radio environment data collected by the sensors or use machine learning for this purpose.

The ATPC algorithm was implemented in the microcontroller, and its performance was tested using RF transceivers using LoRa technology. Based on actual measurements in the radio channel, with the use of a jammer, it was confirmed that the signal strength, i.e., the *k*-factor in the algorithm, affected the accuracy of the effector location in the RSSI method in a real radio channel.

It should be remembered that the power of the transmitter was one of many factors that affected the accuracy of the localization using the RSSI method; others included multipath propagation, obstacles, walls (NLOS [27]), changing the angle of the radiation pattern or the height of the antenna (e.g., in sensors worn by people or animals), the very method of determining RSSI by the RF transceiver (or the appropriate moment of reading after autoregulation by the AGC circuit [34]), or even intentional interference with the radio band. Nevertheless, the presented algorithm can be used along with other distance-estimation error-leveling solutions [20,21,22,23,24,25,26,27,28,29,30], providing a compromise reduction in energy consumption with a small increase in the estimation error for LOS solutions [27].

## Figures and Tables

**Table 1:** Minimum and maximum estimated distance and mean square error for different *k*.

***k*-Factor**	**Min(D)** **[m]**	**Max(D)** **[m]**	**MSE(D)**	**RMSE(D)**
0.0	1.54	4.10	1.40	1.18
0.3	1.72	5.10	1.25	1.12
0.6	2.14	4.10	1.15	1.07
0.9	2.38	4.10	1.10	1.05
1.0	2.96	5.10	0.33	0.58
1.0; no jamming	3.68	5.10	0.06	0.25

## Data Availability

The data presented in this study are available on request from the corresponding author. The data are not publicly available due to project restrictions.
